# Point-of-Care Ultrasound After Medical Screening Exam Reduces Time to Ruptured Ectopic Pregnancy Diagnosis and Surgery

**DOI:** 10.5811/westjem.53932

**Published:** 2026-09-24

**Authors:** Victoria L. Morris, Michael Vu, Yongsu Lee, Evelyn Park, Ravi Soni, Ryan Walsh, Rachel Bower, John C. Waller-Delarosa, Kunal Sharma, Benjamin L. Karfunkle, Richard Gordon

**Affiliations:** * McGovern Medical School at University of Texas Health Science Center Houston, Department of Emergency Medicine, Houston, Texas; † UCLA-Ronald Reagan/Olive View, Olive View, California; ‡ Rush University, Department of Emergency Medicine, Chicago, Illinois

## Abstract

**Introduction:**

Ectopic pregnancy remains the leading cause of first-trimester maternal mortality. Delays in diagnosis and operative management increase morbidity and may be exacerbated by emergency department (ED) crowding. We evaluated whether integration of point-of-care ultrasound (POCUS) into the medical screening exam of pregnant patients—the obstetric medical screening exam—was associated with improved timeliness of care for patients with surgically managed ectopic pregnancy.

**Methods:**

We conducted a single-center retrospective cohort study of adult patients undergoing operative management for ruptured ectopic pregnancy between January 1, 2019, and December 31, 2023, at a county safety-net hospital. The obstetric screening exam was implemented July 12, 2021, and incorporated focused transabdominal pelvic and right upper quadrant ultrasound into the initial ED evaluation, often prior to bed assignment. The primary outcome was time from ED arrival to operating room (OR). Secondary outcomes included time to obstetric consultation, time to hospital admission, ED length of stay (LOS), and POCUS use. Nonparametric testing was used for skewed time data. Logistic regression evaluated the odds of reaching the OR within four hours. Patient-level interrupted time series analysis assessed temporal trends.

**Results:**

A total of 150 patients were included (87 pre- and 63 post-implementation). Median arrival-to-OR time decreased from 516 minutes (interquartile range [IQR] 324–709.5) to 391 minutes (IQR 220–619), a 125-minute reduction (*P* = .020; bootstrap 95% CI, 8–233). The proportion of patients reaching the OR within four hours increased from 16.1% to 31.8% (odds ratio 2.42; 95% CI, 1.11–5.29; *P* = .02). Time to obstetric consultation (210 vs 146 minutes; *P* = .003), time to hospital admission (345 vs 250 minutes; *P* = .005), and ED LOS (451 vs 367 minutes; *P* = .03) were all reduced. Point-of-care ultrasound use increased from 41.4% to 66.7% (*P* < .001), while the remainder underwent radiology ultrasound. Interrupted time series analysis demonstrated no significant immediate change after implementation (*P* = .790), but a significant postintervention trend toward reduced arrival-to-OR time (−0.45 minutes/day, *P* = .01).

**Conclusion:**

Embedding POCUS into the medical screening examination in triage was associated with sustained improvement in operative timeliness for ruptured ectopic pregnancy. Early diagnostic integration within ED workflow may mitigate system-level delays in time-sensitive obstetric emergencies.

## INTRODUCTION

Ectopic pregnancy accounts for approximately 1–2% of all pregnancies and remains the leading cause of maternal mortality in the first trimester, contributing to an estimated 75% of first-trimester deaths and 9–13% of all pregnancy-related deaths.^1^ Despite advances in diagnostic testing and management, mortality rates have remained relatively unchanged in the setting of delays in definitive care.^1^ Tubal rupture, the primary driver of ectopic pregnancy-related mortality, is associated with severe hemorrhage, transfusion, longer hospital length of stay (LOS), and increased morbidity.^2^ Timely diagnosis and management are crucial to reducing morbidity and mortality. However, diagnosis can be challenging, particularly in emergency department (ED) settings where symptoms often overlap with other acute conditions and with prolonged wait times, and where resource constraints may delay critical care.^3^ Further, rupture cannot be reliably predicted by traditional clinical risk factors, gestational age, or serum β–human chorionic gonadotropin (β-hCG) levels. Falcone et al demonstrated that tubal rupture occurred across all β-hCG ranges and was not associated with known ectopic risk factors.^2^ Similarly, discriminatory zone-based strategies and historical predictors have limited ability to identify which pregnancies will progress to rupture or are ruptured.^2^

Young patients with hemorrhage may remain deceptively well appearing during the compensatory phase of shock, with preservation of blood pressure and only subtle abnormalities in routine vital signs. Trauma literature describes this state as occult hypoperfusion—defined as inadequate tissue oxygenation despite normal vital signs—and associates it with worse outcomes. In normotensive trauma patients, the shock index has been shown to identify compensated shock more effectively than blood pressure alone; however, a normal shock index should not be interpreted as reassuring, as compensated shock may persist despite normal heart rate and blood pressure. These physiologic principles are directly relevant to ruptured ectopic pregnancy, in which traditional vital signs correlate poorly with the degree of hemoperitoneum and may fail to identify patients with significant intraabdominal bleeding until compensatory reserve is exhausted and sudden decompensation occurs. Collectively, these findings suggest that reliance on a single set of normal vital signs at triage may contribute to delayed recognition of hemorrhage in this population.^5^–^9^

Timeliness of diagnosis and operative intervention is therefore central to reduce ectopic pregnancy-related morbidity. Delays in care are associated with increased rates of rupture and hemorrhage.^1^ In parallel, a substantial body of literature demonstrates that ED crowding and boarding are associated with delayed treatment, longer hospital LOS, and increased inpatient mortality.^10^,^11^ Even high-acuity patients are not immune to the adverse effects of crowding.^11^ As ED volumes rise and inpatient capacity remains constrained, system-level delays may disproportionately impact time-sensitive conditions such as ruptured ectopic pregnancy.

**Population Health Research Capsule**

What do we already know about this issue?

*Ectopic pregnancy is the leading cause of first-trimester maternal mortality. Emergency department (ED) crowding delays diagnosis, worsening morbidity*.

What was the research question?

*Does embedding ultrasound into the ED medical screening exam reduce time to surgery for ruptured ectopic pregnancy?*

What was the major finding of the study?

*Median arrival-to-OR time decreased 125 min (516 vs 391 min; P = .02; 95% CI, 8–233 min) after implementation of obstetric medical screening exam*.

How does this improve population health?

*Embedding ultrasound at triage can reduce delays for a high-mortality emergency, offering a scalable model in crowded safety-net EDs*.

Within this context, the Emergency Medical Treatment and Labor Act mandates a medical screening examination (MSE) for all patients presenting to the ED. While the MSE serves as a regulatory screening requirement, existing evidence indicates that triage-based evaluations and early physician assessment improve operational flow but do not reliably reduce delays in definitive surgical management in time-sensitive conditions.^1^,^12^ For patients with suspected early pregnancy complications, traditional workflow in crowded ED environments often involves an MSE followed by triage to the waiting room with screening laboratory testing and consultative imaging while the patient waits for a care space in the monitored environment of the ED care area. This sequential approach may introduce diagnostic latency, particularly when imaging availability is constrained by technician staffing or after-hours coverage.^1^

To address these issues, our institution introduced a quality improvement (QI) initiative incorporating point-of-care ultrasound (POCUS) into an obstetric medical screening exam. Prior studies have demonstrated that emergency physicians can accurately diagnose ectopic pregnancy using both transabdominal and transvaginal approaches with POCUS.^3^,^4^ Previous studies also suggest that ED patients with a ruptured ectopic pregnancy who undergo POCUS had shorter times to diagnosis, obstetric consultation, and operating room (OR) arrival compared with those who received radiology-performed ultrasound.^6^–^7^ By embedding POCUS into the triage process, this QI initiative was designed to streamline the diagnosis and treatment of patients with suspected ruptured ectopic pregnancy by reducing time to diagnosis and surgical intervention.

This study evaluates the impact of the obstetric screening exam protocol on the care of patients with ruptured ectopic pregnancy requiring surgical intervention. Specifically, we compared POCUS use rates, time to diagnosis, time to OR arrival, and ED LOS before and after the implementation of the OB MSE.

## METHODS

### Study Design and Setting

This was a single-center retrospective cohort study of all ED patients found to have a ruptured ectopic pregnancy between January 1, 2019–December 31, 2023, at a county safety-net hospital in Houston (average 81,000 visits/year). This facility offers 24/7 consultative pelvic ultrasound via radiology and an in-house obstetrics service with its own triage area staffed by obstetric/gynecologic (OB/GYN) physicians for patients 20 weeks or greater in gestation. Point-of-care ultrasound examinations were performed by a range of clinicians, including physician assistants, residents, ultrasound fellows, and attending physicians with and without ultrasound fellowship training. An obstetric MSE was performed on all patients with known pregnancy or confirmed pregnancy test in the ED.

During the screening exam, clinicians obtained transabdominal transverse and sagittal views of the pelvis to evaluate for presence or absence of intrauterine pregnancy (IUP), and pelvic free fluid. Subsequent to the transabdominal pelvic window, a coronal view of the right upper quadrant was then obtained to evaluate for extrapelvic free peritoneal fluid (Figure 1). Ultrasound images were obtained using 1–5 megahertz (Mhz) curvilinear or 1–5 Mhz phased-array transducers on Sonosite LX, PX, and Xporte systems (Fujifilm, Bothell, WA). All ultrasound images were then reviewed in real time with the emergency attending physician staffing the triage care area at the time.

### Patient Selection

We identified patients at least 18 years of age diagnosed with ectopic pregnancy in the ED based on discharge diagnoses, using *International Classification of Diseases, 10^th^ Revision*, (*ICD-10*) codes. We included all patients who underwent diagnostic laparoscopy for suspected ruptured ectopic pregnancy, regardless of whether POCUS was documented or performed. We excluded patients with nonruptured ectopic pregnancies, intrauterine pregnancies, or pregnancies of unknown location managed without operative intervention, after physician reviewer confirmation of the final diagnosis. There were no cases of heterotopic pregnancy in our sample.

### Data Collection

For retrospective chart review, we followed key methodological recommendations from Worster and Bledsoe to ensure rigor: 1) We defined clear inclusion and exclusion criteria; 2) used a standardized data abstraction form; and 3) defined all key variables a priori. Emergency physicians with specialized fellowship training in POCUS reviewed and extracted all chart values and imaging.^8^ The primary outcomes were as follows; time to diagnosis of ruptured ectopic pregnancy (defined as the interval from ED arrival time to the time a gynecology consult order was placed in the electronic health record (EHR), which occurs simultaneously with verbal communication with the consultant at our institution); time to operative management (the interval from ED arrival to the earlier of either “anesthesia start” time or “arrived in OR” times in the EHR); and ED LOS (defined as the interval from ED arrival to departure from the ED). Time of clinician evaluation was defined as the MSE completion. Time of admission was defined as the time the admit order was placed by the OB/GYN physician. Emergency department LOS, ED admission time, and OR time were all analyzed and recorded separately due to a number of delays affecting the ED. Stable patients would occasionally be admitted to the observation unit or antepartum service prior to going to the OR. Documenting admission times compared to ED LOS allowed us to capture any effect of ED boarding.

We recorded POCUS findings from documented procedure notes detailing the presence or absence of IUP and/or free intraperitoneal fluid. We defined POCUS time as the sooner of the acquisition time or procedure note timestamp, because procedure notes are written in real-time according to the obstetric MSE process. Radiology-performed ultrasound times were defined as the time of acquisition listed on the radiology report. All data were abstracted from the EHR by a team of trained emergency physician reviewers. Because the obstetric MSE occurs on initial evaluation, we recorded triage vitals and calculated mean arterial pressures. We also performed a subanalysis of the primary outcomes OR time and ED LOS for patients with ruptured ectopic pregnancy managed by emergency physicians with and without ultrasound fellowship training.

### Outcome Measures and Analysis

Patients were categorized based on presentation before or after implementation of the obstetric MSE protocol (July 12, 2021). We recorded all time-based outcomes in minutes, including time from ED arrival to ultrasound, obstetrics consultation, hospital admission, OR arrival, and ED LOS. The primary outcome was time from ED arrival to OR. Secondary outcomes included time to obstetrics consultation, time to hospital admission, ED LOS, and the proportion of patients reaching the OR within 240 minutes. All continuous variables are summarized as medians with interquartile ranges (IQR) (Q1–Q3). For continuous outcome variables, the normality assumption was assessed using the Shapiro–Wilk test and quantile-quantile (QQ) plots.

Given non-normal distributions, we performed comparisons between groups using the Wilcoxon rank-sum test. The Welch two-sample *t*-test was conducted as a sensitivity analysis to assess the robustness of the results. We used bootstrap resampling (1,000 iterations) to construct 95% confidence intervals for median differences. We compared categorical outcome variables using the chi-square test. Logistic regression was used to estimate odds ratios (OR) with 95% confidence intervals. The Fisher exact test was performed as a sensitivity analysis. A patient-level interrupted time series (P-ITS) analysis using segmented linear regression was conducted to evaluate temporal trends. The model included elapsed time (days), an indicator for obstetric MSE implementation, and time since implementation. A two-sided *P* value < .05 was considered statistically significant.

## RESULTS

### General

Table 1 displays the observed characteristics of patients presenting before and after implementation of the obstetric MSE. Baseline characteristics were similar between groups, with no statistically significant differences in age, heart rate, gravidity, or estimated blood loss. Mean arterial pressure was higher in the post-implementation group.

Among the 150 patients who required operative intervention for ruptured ectopic pregnancy, 87 presented before and 63 after implementation of the obstetric MSE. Use of POCUS increased from 41.4% before implementation to 66.7% after implementation.

### Primary Outcomes

Median time from ED arrival to OR decreased from 516 minutes (IQR 324–709.5) before the obstetric MSE to 391 minutes (IQR 220–619) after implementation (median difference 125 minutes; *P* = .02). The bootstrap 95% confidence interval for the median difference was 8 to 233 minutes. Results were consistent using the Welch *t*-test (*P* = .02). The proportion of patients reaching the OR within 240 minutes increased from 16.1% before implementation of the obstetric MSE to 31.8% after implementation (*P* = .024). The odds of reaching the OR within 240 minutes were higher after implementation (OR 2.42; 95% CI, 1.11–5.29). The Fisher exact test yielded similar results (*P* = .03).

Table 2 displays each of the primary outcome measures. There was no significant immediate change in time to OR following implementation of the obstetric MSE (β = −27.3 minutes, *P* = .79). However, there was a significant change in slope after implementation (−0.45 minutes/day, *P* = .01), indicating a progressive reduction in time to OR over the study period. Figure 2 displays the distribution of time to OR pre- and post-implementation as a density curve.

### Secondary Outcomes

Time to obstetrics consultation decreased from a median of 210 minutes to 146 minutes (*P* = .003; median difference 64 minutes; 95% CI, 24–125). Time to hospital admission decreased from 345 minutes to 250 minutes (*P* = .005; median difference 95 minutes), although the bootstrap 95% CI (−3 to 173 minutes) included zero. Emergency department LOS decreased from 451 minutes to 367 minutes (*P* = .03; median difference 84 minutes; 95% CI, 6–203). Interrupted time series analysis demonstrated a significant decrease in trend following implementation (−0.35 minutes/day, *P* = .01), shown in Figure 3.

### Point-of-Care Ultrasound Subgroup Analysis

Among patients who underwent POCUS, median time to OR was 324 minutes compared to 533 minutes in those who did not (median difference 209 minutes; *P* < .001; 95% CI, 121.5–307.5). Figure 4 displays the distribution of time as a density curve stratified by POCUS use. Patients who received POCUS were more likely to reach the OR within 240 minutes (37.2% vs 6.5%; OR 7.93; 95% CI, 2.87–21.95; *P* < .001).

## DISCUSSION

In this single-center retrospective cohort study, implementation of an obstetric-focused medical screening examination incorporating POCUS was associated with shorter time to operative management for patients with ruptured ectopic pregnancy. Median time from ED arrival to the OR decreased by 125 minutes, and the proportion of patients reaching the OR within four hours nearly doubled. Time to obstetric consultation, time to hospital admission, and ED LOS were also shorter following implementation, with a concurrent increase in POCUS use.

These findings suggest that integration of ultrasound into the earliest phase of ED evaluation may facilitate earlier recognition of intraperitoneal hemorrhage and accelerate activation of operative care pathways. Importantly, patient-level interrupted time series analysis demonstrated that this improvement was not immediate but occurred progressively over time following implementation, with a significant postintervention slope change of −0.45 minutes per day. This corresponds to an estimated improvement of approximately 41 minutes per quarter and more than two hours per year, suggesting sustained gains in operational efficiency as clinicians adapted to the workflow of the obstetric screening exam.

Prior studies have demonstrated that POCUS can expedite diagnosis in patients with early pregnancy complications and intraabdominal bleeding. However, most prior work has evaluated ultrasound after placement in a treatment bed rather than as part of the initial medical screening examination. Our findings extend this literature by evaluating POCUS as a workflow intervention embedded at the point of triage evaluation. This distinction is clinically meaningful because delays related to bed placement, radiology availability, and patient transport represent known barriers to timely care in crowded EDs. The use of POCUS during the screening exam is not to exclude ectopic pregnancy but rather to evaluate for rupture, positive free fluid to expedite care.

The association between POCUS performance and shorter time to operative intervention further supports the plausibility of this workflow. Patients who underwent POCUS had a median reduction of 209 minutes in time to OR and were significantly more likely to undergo early operative intervention. While this finding does not establish causation, it provides internal consistency suggesting that earlier imaging may contribute to more rapid clinical decision-making. At the same time, the obstetric screening exam represents a combined systems intervention that includes both earlier imaging and workflow restructuring, and the independent contribution of each component cannot be fully isolated.

This study has several strengths. It includes a relatively large cohort of surgically confirmed ruptured ectopic pregnancies and evaluates clinically meaningful, time-based outcomes. The use of nonparametric testing, bootstrap confidence intervals, logistic regression, and patient-level interrupted time series analysis provides a robust and comprehensive analytic framework. In particular, the use of P-ITS strengthens inference by accounting for underlying temporal trends and demonstrating a sustained postintervention improvement rather than a transient effect.

These findings have important clinical implications. Ruptured ectopic pregnancy is a time-sensitive emergency in which delays in diagnosis and operative management may increase hemorrhagic morbidity. In busy EDs, particularly those affected by crowding and boarding, workflows that rely on sequential evaluation and consultative imaging may introduce delays. Integrating focused ultrasound into the medical screening examination may allow earlier identification of patients with suspected hemoperitoneum and facilitate more timely consultation and operative intervention.

Future research should evaluate this approach in multicenter and prospective settings and examine whether improvements in timeliness translate into better patient-centered outcomes, including transfusion requirements, complications, and mortality. Additional work is also needed to determine how similar workflow redesign strategies may be applied to other time-sensitive emergency conditions.

## LIMITATIONS

This study has several limitations. First, its retrospective design introduces the potential for unmeasured confounding and limits the ability to establish causal relationships. Second, the study was conducted at a single high-volume safety-net hospital with an established ultrasound training program, which may limit generalizability to other clinical settings. Third, the protocol for the obstetric screening exam represents a combined intervention involving both earlier use of POCUS and broader workflow changes. As a result, the relative contribution of ultrasound itself versus other system-level factors cannot be independently determined.

Fourth, although patient-level interrupted time series analysis was used to account for underlying temporal trends, residual confounding from concurrent system-level changes cannot be excluded. Implementation of the protocol followed a period of heightened institutional awareness regarding delays in care; other unmeasured changes in clinical practice, staffing, or interdepartmental coordination may have contributed to the observed improvements independent of the intervention. Fifth, identification of POCUS use relied on documentation within the EHR. It is possible that some ultrasound examinations were performed but not documented or archived, commonly referred to as “ghost scans,” which may have resulted in misclassification bias.

Finally, the study period overlapped with the COVID-19 pandemic, which may have influenced ED operations, patient presentation patterns, and hospital throughput in ways that are difficult to fully account for.

## CONCLUSION

Integration of point-of-care ultrasound into the medical screening examination was associated with shorter time to operative management for patients with ruptured ectopic pregnancy, with improvements occurring progressively over time following implementation. These findings suggest that embedding diagnostic ultrasound into ED triage workflow may represent an effective systems-level strategy to reduce delays in time-sensitive obstetric emergencies.

## Figures and Tables

**Figure 1:**
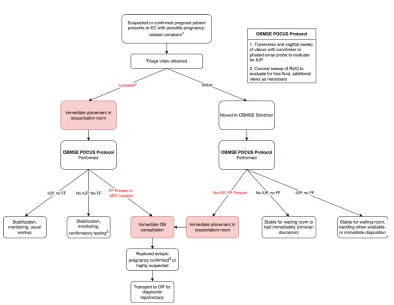
Protocol for obstetric medical screening examination in the emergency department. ^1^Pregnancy-related complaints include confirmed or suspected pregnancy plus one of the following: abdominal pain/cramping, vaginal bleeding, or vaginal discharge. ^2^Unstable vitals include one or more of the following: systolic blood pressure < 90 mm Hg or mean arterial pressure < 65 mm Hg; heart rate > 120 beats per minute; or oxygen saturation < 90% or unreadable. ^3^Confirmatory testing could include one, or a combination of, laboratory serum human chorionic gonadotropic testing; radiology-performed transabdominal and/or transvaginal pelvic ultrasound; computed tomography abdomen/pelvis; or repeat/real-time review of the emergency clinician-performed bedside ultrasound with both the emergency and obstetric attendings present. *EC*, emergency center*; FF*, free fluid*; IUP*, intrauterine pregnancy; *POCUS*, point-of-care ultrasound; *OB*, obstetrics*; OBMSE*, obstetric medical screening exam; *OR*, operating room*; RUQ*, right upper quadrant.

**Figure 2:**
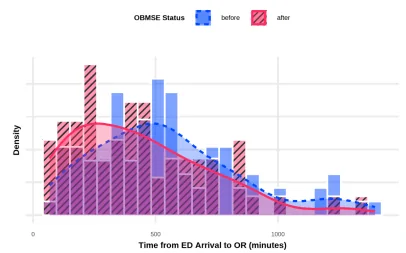
Distributions of time in minutes from emergency department (ED) arrival to before and after implementation of ultrasound in the ED pregnancy screening examination. Curve shows the distribution of the data by smoothing the frequency of observations. It highlights where values are concentrated and allows for easy comparison between groups, even when sample sizes differ. *OBMSE*, obstetric medical screening examination.

**Figure 3:**
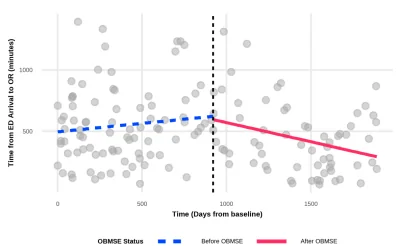
Patient-level interrupted time series analysis demonstrates a significant decrease in trend following implementation of. obstetric medical screening exam. *ED*, emergency department*; OBMSE*, obstetric medical screening exam; *OR*, operating room.

**Figure 4:**
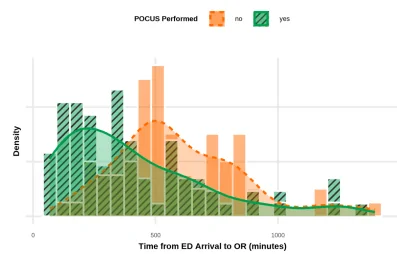
Distributions of time in minutes from emergency department arrival to operating room stratified by point-of-care ultrasound use. *POCUS*, point-of-care ultrasound.

**Table 1:** Characteristics of patients before and after implementation of ultrasound in the emergency department pregnancy screening examination.

	Pre-OBMSE Group (*n* = 87)		Post-OBMSE Group (*n* = 63)		Comparison
	Median	IQR (Q1–Q3)	Median	IQR (Q1–Q3)	*P* value*
Age (years)	30	26–34	33	26–36	.28
Triage HR (bpm)	94	83–102	89	81–98	.15
Triage MAP (mm Hg)	90	83–97	94	88–102	.05
Gravid	3	2–4	3	2–5	.30
EBL (cc)	70	20–250	28	5–300	.78

* P value based on Student *t*-test.

*EBL*, estimated blood loss; *HR*, heart rate; *IQR*, interquartile range; *MAP*, mean arterial blood pressure; *OBMSE*, obstetric medical screening examination; *Q1*, quartile 1; *Q3*, quartile 3.

**Table 2:** Comparison of primary and secondary outcomes before and after implementation of ultrasound in the pregnancy screening examination performed in the emergency department.

	Pre-OBMSE Group (*n* = 87)		Post-OBMSE Group (*n* = 63)		Comparison		
	Median	IQR (Q1–Q3)	Median	IQR (Q1–Q3)	Median Difference	Confidence Interval	*P* value*
Time to Consult (min)	210	141–333.5	146	60.5–245	64	24–125	.003
Time to OR (min)	516	324–709.5	391	220–619	125	8 – 233	.02
ED LOS (min)	451	281–604	367	203–495.5	84	6–203	.03
Time to Admit (min)	345	221.5–132.5	146	132.5–371	95	−3 – 173	.005

* P value based on Mann-Whitney *U* test (or Wilcoxon rank-sum test).

*Dx*, diagnosis; *ED*, emergency department; *IQR*, interquartile range; *LOS*, length of stay; *OBMSE*, obstetric medical screening examination; *OR*, operating room; *Q1*, quartile 1; *Q3*, quartile 3; *time to US*, time to first ultrasound either point of care or radiology.
